# Retraction: S100A8 Contributes to Drug Resistance by Promoting Autophagy in Leukemia Cells

**DOI:** 10.1371/journal.pone.0237489

**Published:** 2020-08-05

**Authors:** The PLOS ONE Editors

Following the publication of this article [1], concerns were raised regarding the western blot results presented in Figs 1A, 1G, 3C, 3E, 4A and 5A.

Specifically,

- The Jurkat band in the S100A8 panel of Fig 1A appears similar to the UT band in the S100A8 (HL60) panel of Fig 1G and the Control band in the S100A8 panel of Fig 3C.
- The HL60/ADR band in the S100A8 panel of Fig 1A appears similar to the MV4-11 band in the S100A8 panel of Fig 1A, the +VCR band in the S100A8 (HL60) panel of Fig 1G, and the +VCR band in the S100A8 panel of Fig 3C.
- The HL60 band in the S100A8 panel of Fig 1A appears similar to the +As2O3 band in the S100A8 (HL60) panel of Fig 1G.
- The K562/A02 band in the S100A8 panel of Fig 1A appears similar to the +ADR band in the S100A8 panel of Fig 3C.
- The K562 band in the S100A8 panel of Fig 1A appears similar to the +ADR band in the S100A8 (HL60) panel of Fig 1G and the UT band in the S100A8 panel of Fig 3C.
- The K562, A02, HL60, and ADR bands in the Actin panel of Fig 1A appear similar to the pLPCX S100A8 control, PI3C3, BECN1 and Atg7 bands in the Actin panel of Fig 3E respectively.
- The bands in the Actin panel of Fig 1G appear similar to the bands in the Actin panel of Fig 3C and the bands in the first four lanes of the Actin panel of Fig 4A, despite representing different experimental conditions in each panel.
- There appear to be vertical lines present between the first and the second lane in the LC3-I/II panel of Fig 3E. In addition, there appear to be horizontal and vertical lines around the bottom band in the fourth lane in the LC3-I/II panel of Fig 3E.
- The band in the first lane of the LC3-I/II panel of Fig 3E appears similar to the band in the first lane of the S100A8 panel of Fig 3E.
- The band in the second lane of the LC3-I/II panel of Fig 3E appears similar to the top band of the fourth lane of the LC3-I/II panel of Fig 3E, and the band in the fourth lane of the S100A8 panel of Fig 3E.
- The band in the third lane of the LC3-I/II panel of Fig 3E appears similar to the band in the second lane of the S100A8 panel of Fig 3E.
- The bands in the fifth, sixth, seventh and eighth lane of the LC3-I/II panel of Fig 3E appear similar to the bands in the fifth, sixth, seventh and eighth lane of the S100A8 panel of Fig 3E.
- There appear to be vertical discrepancies between the bands in the second and the third lane of the ULK1 panel of Fig 5A.

The authors have indicated that these observations are the result of errors in the preparation of the figure files. The authors have provided revised figures to correct the concerns above, but they have not provided the original uncropped figures underlying the blots presented in Fig 1. In addition, the authors have indicated that the original, uncropped images underlying the blots presented in Figs 3, 4 and 5 are no longer available.

In light of the concerns affecting multiple figure panels that question the integrity of these data, the *PLOS ONE* Editors retract this article.

The authors either could not be reached or did not respond to the decision to retract this article.

## References

[1] Yang M, Zeng P, Kang R, Yu Y, Yang L, Tang D, et al (2014) S100A8 Contributes to Drug Resistance by Promoting Autophagy in Leukemia Cells. PLoS ONE 9(5): e97242 10.1371/journal.pone.0097242 24820971 PMC4018274

